# Association between socioeconomic status, learned helplessness, and disease outcome in patients with inflammatory polyarthritis

**DOI:** 10.1002/acr.21677

**Published:** 2012-08

**Authors:** E M Camacho, S M M Verstappen, D P M Symmons

**Affiliations:** Arthritis Research UK Epidemiology Unit, School of Translational Medicine, University of ManchesterManchester, UK

## Abstract

**Objective:**

Independent investigations have shown that socioeconomic status (SES) and learned helplessness (LH) are associated with poor disease outcome in patients with rheumatoid arthritis (RA). Our aim was to investigate the cross-sectional relationship between SES, LH, and disease outcome in patients with recent-onset inflammatory polyarthritis (IP), the broader group of conditions of which RA is the major constituent.

**Methods:**

SES was measured using the Index of Multiple Deprivation 2007 for 553 patients consecutively recruited to the Norfolk Arthritis Register. Patients also completed the Rheumatology Attitudes Index, a measure of LH. SES and LH were investigated as predictors of disease outcome (functional disability [Health Assessment Questionnaire (HAQ)] and disease activity [Disease Activity Score in 28 joints]) in a regression analysis, adjusted for age, sex, and symptom duration. The role of LH in the relationship between SES and disease outcome was then investigated.

**Results:**

Compared to patients of the highest SES, those of the lowest SES had a significantly worse outcome (median difference in HAQ score 0.42; 95% confidence interval [95% CI] 0.08, 0.75). Compared to patients with normal LH, patients with low LH had a significantly better outcome and patients with high LH had a significantly worse outcome (median difference in HAQ score 1.12; 95% CI 0.82, 1.41). There was a significant likelihood that LH mediated the association between SES and disease outcome (*P* = 0.04).

**Conclusion:**

LH is robustly associated with cross-sectional disease outcome in patients with IP, and appears to mediate the relationship between SES and disease outcome. As LH is potentially modifiable, these findings have potential clinical implications.

## INTRODUCTION

It is widely accepted that low socioeconomic status (SES) is associated with a negative impact on physical and mental health (1–4). One example of this is seen in patients with rheumatoid arthritis (RA). A study in Sweden reported that the risk of RA was higher among people of low SES compared to people of high SES (5).

Studies from a number of countries have reported that RA patients of low SES had a worse outcome in terms of disease activity, functional disability, pain, physical and mental health, quality of life, mortality rates, and “RA control” than patients of high SES (6–12). We have previously reported that in a cohort of patients with inflammatory polyarthritis (IP; which includes RA as its major subset), patients of low SES had a worse functional outcome over 3 years of followup than patients of high SES (13).

The mechanism for the relationship between SES and poor health outcome, in general and among patients with RA, is still under debate. There is a growing body of literature that suggests a role for psychosocial factors such as stress and learned helplessness (LH) (14, 15). It has been reported that LH may be associated with poor health outcomes in some people (16). In terms of LH theory (17), the chronic incurable nature of RA may leave patients at risk of feeling out of control. This can then have a negative impact, both mentally (e.g., depression) and behaviorally (e.g., non–self-help behaviors such as noncompliance to prescribed treatment), which can have a negative impact on RA outcome, and thus a detrimental cycle develops.

Among patients with RA, high LH (as measured by the Arthritis Helplessness Index [AHI]) has been shown to be associated with less education, low self-esteem, high anxiety, depression, and greater impairment in activities of daily living (18). High LH (as measured by the more concise Rheumatology Attitudes Index [RAI] [19]) has been found to be associated with more pain, fatigue, and stiffness in RA patients (20).

There is a relative paucity of literature regarding the relationship between social and psychosocial factors and their impact on disease outcome in patients with RA. A recent publication reported that depression in RA patients in California was associated with both SES and disability, between which there was a significant interaction (21), suggesting that depression “moderates” the relationship between disability and SES. Only 1 study has been published that investigated the relationship between SES and LH specifically in terms of their impact upon outcome (5-year mortality) among patients with RA. In that prospective study of 1,348 RA patients in Washington, DC, low SES (as defined by level of formal education) and high LH were both associated with higher mortality (22), although when included in the same statistical model, only LH remained a significant independent predictor of mortality. The authors concluded that the association between SES and mortality was “mediated” by LH. Therefore, there is some evidence of a measureable relationship between SES, LH, and outcome in patients with RA. These 2 studies have suggested different roles for psychosocial factors: moderational and mediational (23). Therefore, further investigation of how these factors may interact is necessary. In addition, further work is needed to determine whether similar observations are replicated in other study populations and with other measures of disease outcome.

Most of the studies cited above have been conducted with patients who have established RA. However, it is also clinically important to consider how social and psychosocial factors are associated with disease outcome in patients with a recent symptom onset. It is difficult to distinguish RA from other similar conditions in the very early stages of the disease. However, patients can easily be classified as having IP, the broader group of diseases of which RA is the major constituent. Therefore, the aim of this investigation of patients with IP was to replicate the observations regarding the relationship between SES and disease outcome, and between LH and disease outcome, reported by previous studies of patients with established RA. Further to this, another aim was to investigate the nature of the role of LH in the relationship between SES and disease outcome in patients with recent-onset IP.

**Significance & Innovations**

- Among patients with recent-onset inflammatory polyarthritis, those of low socioeconomic status (SES) had a worse disease outcome than those of high SES.
- Patients who felt the most helpless had a worse disease outcome than those who felt moderately helpless. Those who reported the lowest level of learned helplessness (LH) had the best disease outcome of all.
- LH appeared to “mediate” the relationship between SES and disease outcome.
- LH may be a modifiable predictor of disease outcome, and therefore these findings have implications for clinicians and patients.

## PATIENTS AND METHODS

### Setting

Patients are invited to join the Norfolk Arthritis Register (NOAR) when they present to a primary care physician or a rheumatologist with recent-onset IP, defined as ≥2 swollen joints that have persisted for ≥4 weeks. A detailed description of the register has been published elsewhere (24).

### Patients

**Figure 1:**
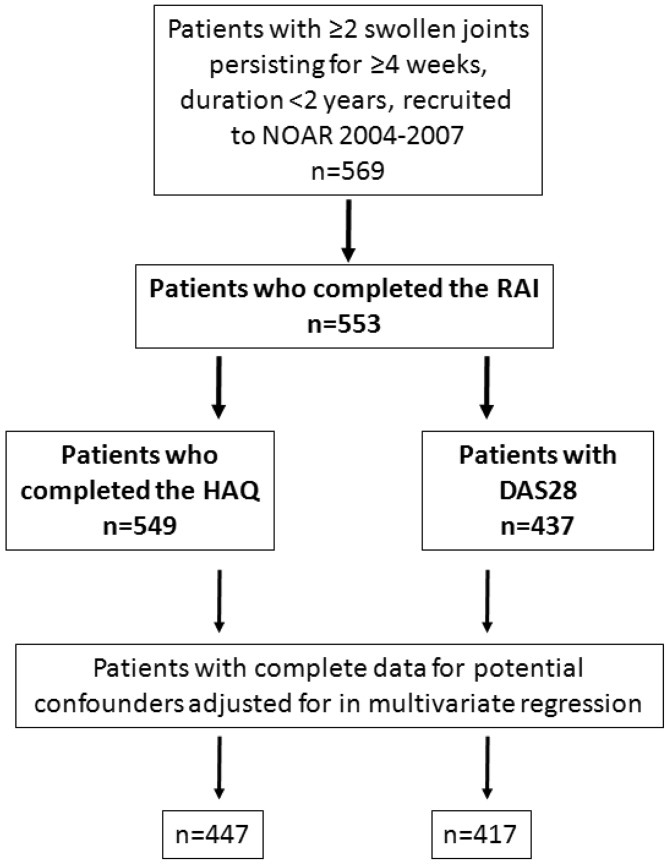
Flow diagram of patients included at each stage of analysis. NOAR = Norfolk Arthritis Register; RAI = Rheumatology Attitudes Index; HAQ = Health Assessment Questionnaire; DAS28 = Disease Activity Score in 28 joints.

A total of 569 patients with a symptom duration of ≤2 years joined the NOAR during 2004–2007. Figure 1 summarizes the number of eligible patients for each part of the analysis described below.

### Data collection

A standardized assessment was carried out by a research nurse and included demographic details, medical history, a joint examination, completion of the British version of the Stanford Health Assessment Questionnaire (HAQ) (25), and patient-reported current and prior IP treatment (steroids or disease-modifying antirheumatic drugs [DMARDs]). Patients reported whether they were current, past, or nonsmokers. A blood sample was taken that was tested for rheumatoid factor (RF; by latex method, positive at a titer of ≥1:40), anti–citrullinated protein antibody (ACPA; by Axis-Shield Diastat kit, positive at >5 units/ml), and C-reactive protein (CRP; by end-point immunoturbidimetric agglutination method, in mg/liter). The Disease Activity Score in 28 joints (DAS28) was calculated, using the method based on the concentration of CRP (26).

### SES

SES was defined as an area-level categorical variable, based on the Index of Multiple Deprivation (IMD) 2007 (27). In the IMD, the UK is divided into “super output areas,” with a minimum population of 1,000 (mean 1,500). Information on income, employment, health, education, barriers to services, crime, and living environment is used to assign a deprivation score to each super output area. These scores are then ranked across the country. For this study, we used postal codes to assign each patient to a nationwide deprivation rank and then to a nationally-determined quartile of deprivation (IMD1 = least deprived, IMD4 = most deprived).

### RAI

**Figure 2:**
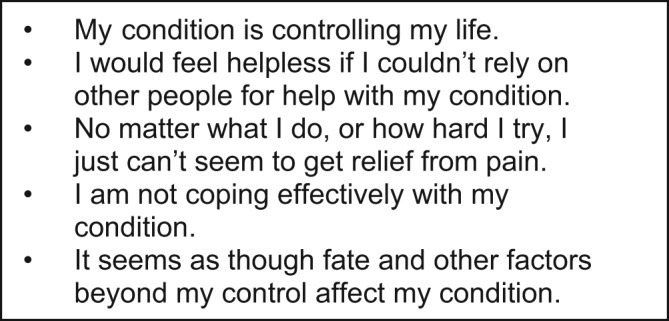
Rheumatology Attitudes Index.

We measured LH using the RAI (Figure 2). This 5-item measure of patients' beliefs about their illness, developed from the longer AHI (18), has been validated as a convenient and acceptable measure of LH for use in research (28). Each item is responded to using a 5-point Likert scale (strongly disagree, disagree, do not agree or disagree, agree, and strongly agree). Total scores range from 5–25, where 5 indicates the fewest feelings of LH. For the analysis presented here, LH was determined as a categorical variable, the levels of which were defined based on findings from the original developers of the AHI (19). The baseline RAI scores of all of the patients included in the analysis here were divided into quartiles, and the middle 2 groups (overall score 9–15) were classified as having “normal” LH, those in the lowest quartile (overall score 5–8) had “low” LH, and those in the highest quartile (overall score 16–25) had “high” LH.

### Statistical analysis

All analyses were carried out using Stata, version 10.1, and were adjusted for sex, age at symptom onset, and symptom duration. Median regression was used to determine the impact of SES (compared to patients from the least deprived areas [IMD1]) and then LH (compared to patients with “normal” LH) on disease outcome (the HAQ and then the DAS28). There was a significant interaction between LH and sex in both of these models; therefore, this was adjusted for in all subsequent models that included LH. These models were then adjusted for additional potential confounders: smoking status, autoantibody status (RF, ACPA), and whether or not the patient had ever received DMARD or steroid treatment. In order to further investigate the interaction between sex and LH, models that included LH as a predictor of disease outcome were then stratified by sex.

The role of LH in the relationship between SES and disease outcome was then explored. The Sobel test (23) was used to determine the extent to which LH (measured as total RAI score [continuous]) “mediated” the relationship between SES (measured as IMD 2007 rank [continuous]) and the 2 disease outcome measures in turn. As an additional test to determine whether LH mediated the relationship between SES and disease outcome, LH was introduced into the regression models predicting disease outcome. A reduction in the statistical significance of the relationship between SES and disease outcome would suggest that LH mediated the relationship. Whether or not LH “moderated” the relationship between SES and disease outcome was determined by adding the interaction between LH and SES to the regression models predicting disease outcome. A statistically significant interaction term would indicate that LH was a moderating factor in the relationship between SES and disease outcome (23).

Finally, a crude test was carried out to determine whether the relationships observed were likely to be different among patients who met the 1987 American College of Rheumatology (ACR) criteria for RA at baseline and those who did not (29). An interaction between SES and a binary variable describing whether or not patients met the criteria was added to the regression models predicting disease outcome.

## RESULTS

### Cohort characteristics

A total of 553 patients completed the RAI at baseline. As defined above, 274 (49.5%) had normal LH, 131 (23.7%) had low LH, and 148 (26.8%) had high LH. The median age at symptom onset was 57.2 years (interquartile range [IQR] 46.4–68.1 years). Three hundred forty-seven patients (62.8%) were women. The median symptom duration at baseline was 5 months (IQR 3–10 months). The cohort characteristics at baseline are shown in Table 1. This cohort of patients was less deprived than the UK national average because 66% of patients were in the top 2 quartiles of SES.

**Table 1:** Cohort characteristics at baseline (n = 553)*

	Value
Age at IP onset, median (IQR) years	57.2 (46.4–68.1)
Female sex	347 (62.8)
Symptom duration, median (IQR) months	5 (3–10)
Smoking status (n = 551)	
Current smoker	119 (21.6)
Past smoker	233 (42.3)
Nonsmoker	199 (36.1)
Met 1987 ACR criteria for RA	253 (45.8)
Positive for RF (n = 518)	254 (49.0)
Positive for ACPAs (n = 453)	159 (35.1)
HAQ score (n = 549), median (IQR)	0.88 (0.38–1.50)
DAS28 score (n = 437), median (IQR)	3.82 (2.94–4.82)
Socioeconomic status†	
IMD1 (least deprived)	154 (27.9)
IMD2	211 (38.1)
IMD3	121 (21.9)
IMD4 (most deprived)	67 (12.1)
Learned helplessness‡	
Low	131 (23.7)
Normal	274 (49.5)
High	148 (26.8)

* Values are the number (percentage) unless otherwise indicated. IP = inflammatory polyarthritis; IQR = interquartile range; ACR = American College of Rheumatology; RA = rheumatoid arthritis; RF = rheumatoid factor; ACPAs = anti–citrullinated protein antibodies; HAQ = Health Assessment Questionnaire; DAS28 = Disease Activity Score in 28 joints.

† Based on nationwide Index of Multiple Deprivation (IMD) 2007 rank.

‡ As measured by the Rheumatology Attitudes Index (low = 5–8, normal = 9–15, and high = 16–25).

### Cohort characteristics and LH

The cohort characteristics are shown by baseline LH in Table 2. Patients who had low LH were the oldest at symptom onset (median 60.9 years), and those who had high LH were the youngest at symptom onset (median 52.5 years). The percentage of female patients was lowest in the low LH group (55.0%).

**Table 2:** Cohort characteristics at baseline, by baseline LH*

	Low LH (n = 131)	Normal LH (n = 274)	High LH (n = 148)
Age at IP onset, median (IQR) years	60.9 (48.2–71.2)	58.6 (48.1–68.9)	52.5 (41.0–63.4)
Female sex	72/131 (55.0)	182/274 (66.4)	93/148 (62.8)
Socioeconomic status†			
IMD1 (least deprived)	39/131 (29.7)	80/274 (29.2)	35/148 (23.7)
IMD2	44/131 (33.6)	115/274 (42.0)	52/148 (35.1)
IMD3	28/131 (21.4)	56/274 (20.4)	37/148 (25.0)
IMD4 (most deprived)	20/131 (15.3)	23/274 (8.4)	24/148 (16.2)
Symptom duration, median (IQR) months	5 (3–8)	5 (3–10)	6 (3–11)
Smoking status			
Current smoker	30/130 (23.1)	45/273 (16.5)	44/148 (29.8)
Past smoker	55/130 (42.3)	122/273 (44.7)	56/148 (37.8)
Nonsmoker	45/130 (34.6)	106/273 (38.8)	48/148 (32.4)
Met 1987 ACR criteria for RA	41/131 (31.3)	128/274 (46.7)	84/148 (56.8)
Positive for RF	57/121 (47.1)	130/263 (49.4)	67/134 (50.0)
Positive for ACPAs	30/100 (30.0)	85/235 (36.2)	44/118 (37.3)

* Values are the number/total (percentage) unless otherwise indicated. Learned helplessness (LH) was measured by the Rheumatology Attitudes Index (low = 5–8, normal = 9–15, and high = 16–25). IP = inflammatory polyarthritis; IQR = interquartile range; ACR = American College of Rheumatology; RA = rheumatoid arthritis; RF = rheumatoid factor; ACPAs = anti–citrullinated protein antibodies.

† Based on nationwide Index of Multiple Deprivation (IMD) 2007 rank.

High LH was found least commonly (23.7%) in patients of the highest SES (IMD1), whereas the proportion of patients from the lowest level of SES (IMD4) was smallest in the normal LH group (8.4%). The proportion of current smokers was highest among patients with high LH (29.8%). There appeared to be a trend between LH and meeting the 1987 ACR criteria for RA, i.e., the proportion of patients meeting the criteria was highest in the high LH group and lowest in the low LH group. The same trend was observed for patients who were positive for ACPA. In other words, high LH was associated with markers of more severe disease.

All subsequent results are from analyses adjusted for sex, age at symptom onset, and symptom duration, unless otherwise stated.

### SES and disease outcome

The relationship between SES and disease outcome is shown in Table 3. Patients of the lowest SES (IMD4) had the highest median HAQ and DAS28 scores. There appeared to be a threshold effect of SES on disease outcome; patients in the highest 2 quartiles had a better disease outcome than patients in the lowest quartile of SES. Compared to people of the highest SES (IMD1), those of the lowest SES had significantly higher HAQ scores on average (median difference 0.42; 95% confidence interval [95% CI] 0.08, 0.75). Compared to people in IMD1, those in IMD4 also had a significantly higher DAS28 score (median difference 0.64; 95% CI 0.14, 1.14). Adjustment for additional potential confounders revealed another significant difference: the difference in HAQ score of patients in IMD3 compared to those of the highest SES (median difference 0.28; 95% CI 0.13, 0.44). The difference in the DAS28 score between patients of the highest and lowest SES was no longer significant following these additional adjustments (median difference 0.38; 95% CI −0.16, 0.93).

**Table 3:** Summary of the impact of SES and LH on disease outcome (HAQ and DAS28 scores) at baseline*

	HAQ score (n = 549)	DAS28 score (n = 437)
SES		
Median (IQR) (n)		
IMD1	0.88 (0.25–1.38) (153)	3.69 (2.94–4.68) (125)
IMD2	0.88 (0.38–1.50) (211)	3.77 (2.77–4.63) (161)
IMD3	0.88 (0.38–1.63) (119)	3.93 (3.05–4.83) (99)
IMD4	1.19 (0.38–1.63) (66)	4.14 (3.05–5.08) (52)
Median difference (95% CI) vs. IMD1†		
IMD2	−0.02 (−0.26, 0.23)	0.14 (−0.23, 0.50)
IMD3	0.09 (−0.19, 0.37)	0.26 (−0.15, 0.67)
IMD4	0.42 (0.08, 0.75)‡	0.64 (0.14, 1.14)‡
Median difference (95% CI) vs. IMD1§		
IMD2	0.07 (−0.07, 0.20)	−0.03 (−0.41, 0.35)
IMD3	0.28 (0.13, 0.44)‡	0.19 (−0.24, 0.63)
IMD4	0.46 (0.27, 0.65)‡	0.38 (−0.16, 0.93)
Median difference (95% CI) vs. IMD1¶		
IMD2	0.03 (−0.15, 0.21)	−0.07 (−0.38, 0.25)
IMD3	0.06 (−0.15, 0.27)	0.06 (−0.30, 0.42)
IMD4	0.02 (−0.23, 0.27)	0.34 (−0.10, 0.78)
*P*	0.96	0.31
LH		
Median (IQR) (n)		
Low	0.25 (0–0.63) (130)	2.90 (2.39–3.61) (89)
Normal	0.88 (0.38–1.38) (272)	3.86 (2.98–4.76) (230)
High	1.63 (1.0–2.0) (147)	4.57 (3.48–4.49) (118)
Median difference (95% CI) vs. normal†		
Low	−0.39 (−0.69, −0.10)#	−0.89 (−1.39, −0.40)#
High	1.12 (0.82, 1.41)#	1.23 (0.74, 1.72)#
Median difference (95% CI) vs. normal§		
Low	−0.38 (−0.66, −0.10)#	−0.68 (−1.23, −0.14)#
High	1.05 (0.75, 1.34)#	1.03 (0.48, 1.59)#

* SES = socioeconomic status; LH = learned helplessness; HAQ = Health Assessment Questionnaire; DAS28 = Disease Activity Score in 28 joints; IQR = interquartile range; IMD1 = Index of Multiple Deprivation rank 1 (least deprived); IMD4 = IMD rank 4 (most deprived); 95% CI = 95% confidence interval.

† Adjusted for age at symptom onset, symptom duration, and sex (HAQ, n = 549; DAS28, n = 437).

‡ Significant difference compared to patients from IMD1.

§ Adjusted for age at symptom onset, symptom duration, sex, smoking status, rheumatoid factor status, anti–citrullinated protein antibody status, and treatment with disease-modifying antirheumatic drugs or steroids (HAQ, n = 447; DAS28, n = 417).

¶ Regression model including both SES and LH. Adjusted for age at symptom onset, symptom duration, sex, and LH.

# Significant difference compared to patients with normal LH.

### LH and disease outcome

The relationship between LH and disease outcome is also shown in Table 3. The median HAQ and DAS28 scores showed a clear increase with increasing LH (low to high). Compared to patients with normal LH, those with low LH had significantly lower baseline HAQ scores on average (median difference −0.39; 95% CI −0.69, −0.10), and those with high LH had significantly higher HAQ scores (median difference 1.12; 95% CI 0.82, 1.41). Compared to patients with normal LH, those with low LH also had a significantly lower DAS28 score (median difference −0.89; 95% CI −1.39, −0.40), and those with high LH had a significantly higher DAS28 score (median difference 1.23; 95% CI 0.74, 1.72). Adjustment for additional potential confounders had little impact on the median differences in HAQ score by LH, and had a modest impact on the size of the difference in DAS28 score, although the statistical significance and direction of the effects remained unchanged.

### Sex, LH, and disease outcome

The relationships between LH and disease outcome in the 2 sexes are shown in Table 4. The values for the entire cohort reported in Table 4 are unadjusted for sex and therefore different from the values reported in Table 3. The effect of LH on disease outcome was generally more pronounced in male patients than in female patients.

**Table 4:** Relationship between LH and disease outcome (HAQ and DAS28 scores) and investigation of the role of LH in the relationship between SES and disease outcome*

	Entire cohort (n = 549)	Female patients (n = 344)	Male patients (n = 205)
HAQ score			
Low LH vs. normal LH, median difference (95% CI) vs. normal LH†	−0.55 (−0.70, −0.39)‡	−0.60 (−0.82, −0.37)‡	−0.34 (−0.65, −0.03)‡
High LH vs. normal LH, median difference (95% CI) vs. normal LH†	0.79 (0.64, 0.95)‡	0.67 (0.46, 0.88)‡	1.17 (0.86, 1.49)‡
Sobel test statistic	−2.04§	−2.02§	−0.67
SES → HAQ score mediated by LH, %	49	55	33
*P*	0.04	0.04	0.50
Statistical significance of SES × LH interaction, *P*†	0.82	0.84	0.85
DAS28 score			
Low LH vs. normal LH, median difference (95% CI) vs. normal LH†	−0.97 (−1.35, −0.59)‡	−0.59 (−1.01, −0.17)‡	−0.94 (−1.61, −0.26)‡
N	437	287	150
Low LH vs. normal LH, median difference (95% CI) vs. normal LH†	0.66 (0.32, 1.01)‡	0.57 (0.20, 0.94)‡	1.34 (0.66, 2.01)‡
Sobel test statistic	−2.10§	−1.97§	−0.66
SES → DAS28 score mediated by LH, %	64	65	51
*P*	0.04	< 0.05	0.51
Statistical significance of SES × LH interaction, *P*†	0.63	0.61	0.06

* LH = learned helplessness; HAQ = Health Assessment Questionnaire; DAS28 = Disease Activity Score in 28 joints; SES = socioeconomic status; 95% CI = 95% confidence interval.

† Adjusted for age at symptom onset and symptom duration, but not adjusted for sex.

‡ Significant difference compared to patients with normal LH.

§ LH is a significant mediator of the relationship between SES and HAQ/DAS28 scores.

### Role of LH in the relationship between SES and disease outcome

The results of the Sobel test of mediation are shown in Table 4, for the entire cohort and stratified by sex. LH mediated a significant proportion of the relationship between SES and disease outcome; this was the case for HAQ score (49% mediated; *P* = 0.04) and the DAS28 score (64% mediated; *P* = 0.04). The results of the Sobel test were similar among female patients. However, according to the Sobel test, LH was not a significant mediator among male patients.

When LH and SES were included in the same regression model, the relationship between SES and the HAQ score became statistically nonsignificant (*P* = 0.96) (Table 3), suggesting that LH mediates the relationship between SES and disease outcome. The same was true for the DAS28 score (*P* = 0.31). The same pattern was observed when the regression models were stratified by sex (data not shown).

As shown in Table 4, the interaction between SES and LH was not significant in the model predicting the HAQ score for the entire cohort, or for either sex. The same was true for the DAS28 score, although among male patients the interaction term was bordering on statistical significance (*P* = 0.06). This suggests that overall LH does not moderate the relationship between SES and disease outcome, although it is possible that the role of LH in the relationship between SES and disease outcome may be different in men and women.

### ACR criteria for RA

The interaction between SES and whether or not patients met the 1987 ACR criteria for RA was not significant in the model predicting the HAQ score (*P* = 0.46) or the DAS28 score (*P* = 0.52). The interaction between LH and whether or not patients met the 1987 ACR criteria for RA was also not significant in the model predicting the HAQ score (*P* = 0.15) or the DAS28 score (*P* = 0.34). This suggests that the observations reported here for patients with “definite” RA are not different from those with undifferentiated IP.

## DISCUSSION

SES was significantly associated with disease outcome in our cohort of patients with recent-onset IP; patients of low SES had a worse outcome than patients of high SES. LH was robustly associated with disease outcome; patients with high LH had higher HAQ and DAS28 scores than those with normal LH, and patients who had the lowest LH had the best outcome of all. The association between SES and disease outcome was no longer statistically significant when adjusted for LH, which along with the results of the Sobel test suggests that LH mediates the relationship between SES and disease outcome. It does not appear that LH moderates the relationship between SES and disease outcome, although there is some evidence of a sex difference in the way that LH behaves.

This is the first study to investigate the relationship between LH, SES, and disease outcome together in patients with recent-onset IP. Our patients were recruited shortly after symptom onset from primary and secondary care, and so the cohort includes patients with a range of baseline disease severity. Seventy-five percent of NOAR patients have been shown to fulfill the 1987 ACR criteria for RA cumulatively within 5 years of symptom onset (30). The median symptom duration of this cohort was only 5 months, and so the application of the criteria for RA at baseline is of limited validity; it is not possible to say so early in the disease course which patients will go on to develop RA. With this in mind, the most relevant existing literature relates to patients with RA.

Our results reinforce previous reports that RA patients of low SES have a poorer outcome than those of higher SES (6–10), which we have also previously reported in the NOAR (13). Together with the finding that RA is more prevalent in people of low SES (5), this highlights the need to ensure that patients from low SES backgrounds are afforded the same opportunities to receive the appropriate level of care as other patients. What we have shown here also supports findings that high LH is associated with an unfavorable outcome in patients with RA (18–20). Our findings also mirror the prospective study of LH and SES in more than 1,000 RA patients in the US, which reported that high LH and low SES were univariately associated with higher mortality during the study period (22).

A strength of this investigation is that the RAI is a validated measure of LH in patients with arthritis (28), and its concise nature is conducive to a high completion rate (97% of patients in this cohort completed the RAI at baseline). A limitation of this investigation was the use of a postal code–based measure of SES. Although the IMD works at a level where “areas” contain a minimum of 1,000 residents, there is generally a broad mix of house type in most residential areas in the UK, and so SES may be overestimated for some and underestimated for others in the same area: the “ecological fallacy.” The IMD was developed to improve on measures of SES such as the Townsend Index, which does not account for differences in rural and urban measures of SES. The NOAR study region covers a large geographic area comprising both rural and urban areas, and so the IMD is a more appropriate measure of area-level SES in this context (13). Individual-level measures of SES such as income can also present problems in this setting because patients are often unwilling to disclose their income. Additionally, personal income or occupation as a measure of standard of living does not allow for factors such as spousal income or inheritance, which may afford a high SES alongside low personal income. The IMD was chosen over occupation as the indicator of SES in this analysis because data were available for all of the patients.

Another limitation of this study is that the geographic area from which NOAR patients are recruited is not completely representative of the UK as a whole. The levels of deprivation used in this analysis represent nationwide quartiles, i.e., ∼25% of the population at each level. However, in this cohort there is an overrepresentation (38%) of people in the second least deprived group (IMD2) and an underrepresentation (12%) of people in the most deprived group (IMD4). Another disadvantage of this study design is that, because it cross-sectional, it is not possible to infer directionality of the relationship between LH and disease outcome. Further investigation of the impact of LH on subsequent disease outcome is required. Future work is also needed to further explore the role of LH in the relationship between SES and disease outcome, since only 2 possibilities were considered here.

It is likely that the mechanisms involved in the association between SES, LH, and disease outcome are multilevel. A widely accepted reason for the association between SES and poor health outcomes is access to health care (12). All of the patients recruited to the NOAR are under the care of an NHS physician, and so SES should not impact access to care. However, it is possible that other factors associated with SES, perhaps level of education, may enable patients of higher SES to have more beneficial interactions with health care professionals (13), for example, feeling able to express concerns if they feel that their care plan is not appropriate. Alternatively, patients who live in more deprived areas may be subject to more negative environmental factors, which may have a detrimental effect on health in general or may lead to more comorbid diseases.

The relationship between LH and disease outcome is more complex, especially with respect to causality. LH has been described as an “attributional style” (20); it is possible that patients who have a “can do” philosophy to their disease may be more likely to actively try to improve their disease outcome, for example, by losing weight or generally maintaining a healthy lifestyle. This may then be reflected in patients' responses on the HAQ, which can be viewed as a measure of physical helplessness. However, it is also understandable that patients with extremely disabling or active disease may already feel very helpless or frustrated. LH may also have an impact on disease outcome if patients with high LH are less compliant with medication, although we were unable to investigate this here.

It has been suggested that LH is associated with a passive style of coping (31), something that people of high SES may have more opportunity to overcome, through education or along the path to well-paid, but often highly stressful, occupations. It has also been suggested that patients of low literacy feel more helpless (20); presumably, literacy rates are highly correlated with SES. Alternatively, if LH itself is seen as a measure of disease outcome, then SES may be associated with measures of LH in the same way as measures of physical disease outcome.

SES is a complex indicator of a number of factors that may influence disease outcome, such as demographic or genetic factors (22). Our findings suggest that LH is a measure of the psychological or behavioral component of the relationship between SES and disease outcome in patients with IP. There may be a role for behavior-change interventions, especially those focused on providing information and facilitation of goal setting, that have specifically been found to be helpful among people of low SES in the general population (32). Patients with IP may be helped by learning to put in place their own coping mechanisms and to recognize that their actions and behaviors can have a positive impact on their health.

This investigation supports previous findings that SES and LH are associated with disease outcome in patients with RA. Existing knowledge has been built upon here by identifying that LH appears to mediate the relationship between SES and disease outcome in patients with early IP. Unlike SES, LH is a potentially modifiable factor that is reliably associated with disease outcome. Therefore, clinicians should acknowledge and address feelings of LH in their patients.
